# The Role of Meteorological Variables and Aerosols in the Transmission of COVID‐19 During Harmattan Season

**DOI:** 10.1029/2021GH000521

**Published:** 2022-02-01

**Authors:** S. Ogunjo, O. Olaniyan, C.F. Olusegun, F. Kayode, D. Okoh, G. Jenkins

**Affiliations:** ^1^ Department of Physics Federal University of Technology Akure Nigeria; ^2^ National Weather Forecasting and Climate Research Centre Nigerian Meteorological Agency Abuja Nigeria; ^3^ Centre for Atmospheric Research National Space Research and Development Agency Kogi State University Campus Anyigba Nigeria; ^4^ Department of Meteorology and Atmospheric Sciences Penn State University University Park PA USA

## Abstract

The role of atmospheric parameters and aerosols in the transmission of COVID‐19 within tropical Africa, especially during the harmattan season, has been under‐investigated in published papers. The harmattan season within the West African region is associated with significant dust incursion from the Bodele depression and biomass burning. In this study, the correlation between atmospheric parameters (temperature and humidity) and aerosols with COVID‐19 cases and fatalities within seven locations in tropical Nigeria during the harmattan period was investigated. COVID‐19 infection cases were found to be significantly positively correlated with atmospheric parameters (temperature and humidity) in the southern part of the country while the number of fatalities showed weaker significant correlation with particulate matters only in three locations. The significant correlation values were found to be between 0.22 and 0.48 for particulate matter and −0.19 to −0.32 for atmospheric parameters. Although, temperature and humidity showed negative correlations in some locations, the impact is smaller compared to particulate matter. In December, COVID‐19 cases in all locations showed strong correlation with particulate matter except in Kano State. It is suggested that a reduction in atmospheric particulate matter can be used as a control measure for the spread of COVID‐19.

## Key Points

- The role of meteorological parameters and particulate matters in the number of COVID‐19 cases varies with location within Nigeria
- Local climate affects the relationship between particulate matter and the number of infected persons
- The role of socio‐economic, demographic, and environmental factors in the spread of COVID‐19 were discussed

## 1 Introduction

The outbreak of severe acute respiratory syndrome coronavirus 2 (SARS‐CoV‐2), originating from the Wuhan region of China, has caused devastating damages across the world. The impact of SARS‐CoV‐2 (COVID‐19) has been reported in global transportation (Abu‐Rayash & Dincer, 2020) and power system (Gulati et al., 2021). The greatest impact has been on the global health system, overwhelming the health care facilities of many developed and developing countries (Armocida et al., 2020). COVID‐19 infections could be asymptomatic, symptomatic or presymptomatic leading to severe forms which require intubation and can lead to death (Lee et al., 2020). For instance, older patients with more than one underlying medical condition (e.g., hypertension, obesity, diabetes, chronic kidney diseases etc.) are more predisposed to severe COVID‐19 complications (Fang et al., 2020; R. T. CDC et al., 2020), although younger and healthier patients are more likely to respond faster to treatment (C. CDC et al., 2020; Wang et al., 2020). Multiple organ failure and cardiopulmonary complications, such as myopericarditis, pulmonary embolism and acute respiratory distress syndrome, represent some of the major complications of severe COVID‐19. The COVID‐19 outbreak has created a global health crisis. There are a total of 171,259,456 COVID‐19 cases, 3,567,030 deaths worldwide as of 2 June 2021 (John Hopkins University, 2021) with Nigeria having the highest number of cases (166,534) and deaths (2,099) in West Africa. The first case of COVID‐19 incidence was reported on 27 February 2020. Since then the reported confirmed cases increased considerably to about 1,350 cases before the end of the second month. Lagos State, Kano State and Federal Capital Territory (FCT) reported the highest number of cases in Nigeria. The Federal government of Nigeria and various State Governments imposed restrictive measures such suspension of all activities and religious gatherings, indefinite closure of public and private schools/institutions, extension of the travel ban to some countries, suspension of the operation of Nigerian Railway Corporation, closing of borders, shops, markets, motor parks, offices, restriction of intra‐states and inter‐states movements within the country (Ayinde et al., 2020).

Non‐pharmaceutical interventions in COVID‐19 cases have been found to improve air quality in many countries across the world (Fuwape et al., 2021; Gautam, 2020). Air pollution has been shown to reduce respiratory resistance against bacterial and viral infections (Ciencewicki & Jaspers, 2007; Dominici et al., 2006). There are emerging proofs that people living in areas with poor air quality might be more frequently infected by COVID‐19 (references). Some of the countries that were greatly affected are those with a poor air quality index, such as China (Zhang et al., 2020), Italy (Zoran et al., 2020), and United States (Pavilonis et al., 2021). Considering Northern Italy, particularly Lombardy and Emilia Romagna are among the most polluted areas in Europe. 12% mortality rate was recorded in those places compared to some other parts of Italy that recorded 4.5% mortality (Conticini et al., 2020). A study conducted in Germany showed that environmental pollution and the COVID‐19 pandemic are significantly connected: higher levels of local air pollution increase the number of deaths of COVID‐19, leading to a wider spread of the virus (Isphording & Pestel, 2021). Similar conclusions have been made for three French cities using an innovative machine learning approach (Magazzino et al., 2020).

In Africa, the factors contributing to poor air quality include: boom in industrialization, surge in the population growth, dust sources, use of non‐renewable energy sources for cooking and heating, and poor waste management (Amegah & Agyei‐Mensah, 2017; Lacey et al., 2017). Du and Li (2016) also showed that increased PM2.5 pollution is related to increased respiratory infection and diseases as well as cardiovascular and circulatory diseases. For instance, Lacey et al. (2017) linked anthropogenic emissions from residential activities to more than 13,000 annual premature deaths in Africa. They also projected more than 78,000 annual premature deaths by the year 2030 due to the health impacts of particulate matter concentration. According to Han et al. (2017), in the year 2010, 30% of people from Western Africa, approximately 86 million, were exposed to fine inhalable particulate matter (PM2.5) pollution. The dominant culprit for the poor air quality in Africa is PM2.5, which is a conglomerate of chemicals (inorganic or organic) and compounds such as dust, hydrocarbons, cooking stoves, fires from bush burning etc. Similarly, the presence of PM10 contributes to respiratory illness, lung and premature death in sensitive individuals (EPA, 2009). Although, it is difficult to directly link mortality rates to air pollution due to a lack of information on the toxicity of the particles from the various sources of the pollutant. The non‐availability of continuous long‐term data over the region due to the sparse network of air quality observing stations poses another form of restraint. However, in recent times, the increased network of air quality monitoring stations across West Africa could provide additional robust information on the role of air pollution and atmospheric parameters in the spread of COVID‐19 across the region during the harmattan with the purpose of communicating associated risks. In addition to other sources of pollution, desert dust and biomass burning are important seasonal sources of particulate matter. Desert dust and particulate matter emissions may be drivers for transmissible and non‐transmissible respiratory disease and a leading cause of infant mortality and premature death in Africa (Bauer et al., 2019; Heft‐Neal et al., 2018). Desert dust can have harmful effects on the respiratory, cardiovascular, and cerebrovascular systems (De Longueville et al., 2013; Goudie, 2014). Toure et al. (2019) showed that there are many asthma cases and acute respiratory infections in Senegal throughout the year. Further, Marone et al. (2020) show that bacteria exists on the surface of dust particles, with some serving as pathogens that could have health impacts in West Africa during the winter and spring.

Nigeria has a tropical climate with variable wet and dry seasons, depending on location. It is wet most of the year in the south but mostly dry in the north. The wet season lasts from March to November in the south, however in the far north it lasts only from mid‐May to September. The dry season is usually referred to as the harmattan season. Harmattan, is a cool dry wind that blows from the northeast or east in the western Sahara. It usually carries large amounts of dust from the Sahara Desert which can be transported as far as the Gulf of Guinea. The Sahara Desert is responsible for up to 50% of major global dust emissions (Luo et al., 2003; Washington et al., 2003) and it is one of the major sources of aerosol pollutants during the harmattan season in Sub Saharan Africa (Washington & Todd, 2005). In Nigeria, the northern parts usually experience higher frequency of occurrence of very low horizontal visibility compared to the southern part due its nearness to the Saharan dust source (Ochei & Adenola, 2018). The harmattan wind is usually caused by large‐scale circulations over the Bodele region initiated by the intensification of the Azores Subtropical high‐pressure system during the boreal winter. The circulation which include the ridging of the Libyan High and pulsing of the pressure gradient is responsible for the lifting of large amounts of dust. The lifted dust is propagated by the northeasterlies in which Low Level Jet at 925 hPa is embedded (Washington et al., 2003). The dust is usually located northward of the Inter‐tropical discontinuity (ITD) which is the zone of convergence between the moist south westerly trade wind from the South Atlantic Ocean and the dry northeasterly trade wind from the Sahara Desert. The ridging effect of the Libyan high pushes the ITD southwards, sometimes as far as the Gulf of Guinea. Studies have shown that the majority of dust affecting Nigeria is generally raised mainly from the Bodele depression (Todd et al., 2007). The extent of the dust from the Bodele depression has been found to extend to the Amazon basin (Ben‐Ami et al., 2010). The aerosols carried by the harmattan wind consist of particulate matter (PM 10 and 2.5) that are small enough to be inhaled into the deepest parts of the lung, PM 2.5 is especially detrimental to health. The harmattan season is usually accompanied with dryness with humidity, dropping as low as 10% in severe cases which can result in spontaneous nosebleeds for some people. It leads to conditions such as dried skin, dried and chapped lips and dehydration. It also affects the respiratory system as people experience difficulty in breathing, aggravation of asthma and easy transmission of respiratory diseases like cough, catarrh, and tuberculosis (Sufiyan et al., 2020). The season is usually associated with increase in hospitalization for congestive cardiac failure and cerebrovascular accident (Okeahialam, 2016).

**Figure 1:**
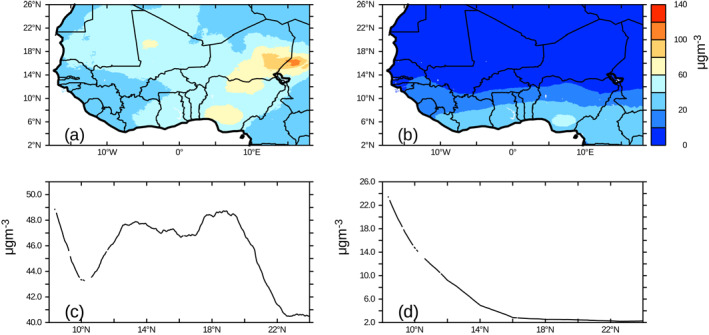
PM2.5 horizontal distribution (a–b) and latitudinal variation (c–d) averaged over 10°W to 10°E across West Africa. LHS shows PM2.5 with Dust and Sea Salt and RHS shows PM2.5 without Dust and Seasalt.

The lack of in situ particulate matter data in Africa requires that satellite and global chemical transport models are used to estimate particulate matter in Africa (Bauer et al., 2019; Van Donkelaar et al., 2015). Figure 1a shows the annual PM2.5 concentrations across West Africa averaged for 1998–2018 without sea salt and dust aerosols. There is a strong north‐south gradient in Figure 1a across Africa suggestive of greater anthropogenic sources towards the Gulf of Guinea. The inclusion of dust in particular show that annual PM2.5 concentrations are considerably higher than the WHO recommended values of PM2.5 concentrations of 10 μg/m^3^ (Figure 1b). The latitudinal PM2.5 concentrations shows that without dust and sea salt the highest concentrations are found near the coast and decrease rapidly with latitude (Figure 1c). On the other hand, when dust and sea salt are included, a multi‐modal latitudinal distribution of PM2.5 concentrations are found with peaks found near the Gulf of Guinea, near 12°N and 19°N (Figure 1d).

**Figure 2:**
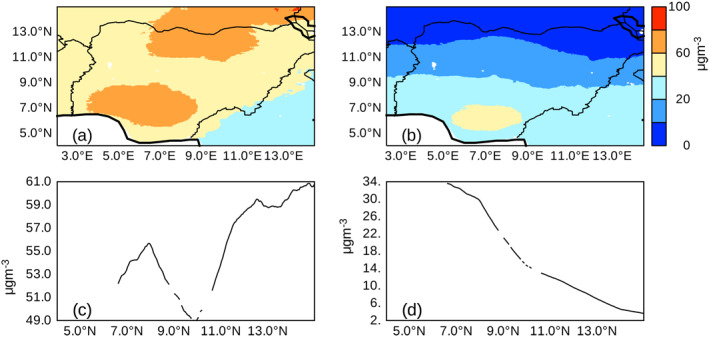
PM2.5 horizontal distribution (a–b) and latitudinal variation (c–d) averaged over 3°E to 14°E across Nigeria. LHS shows PM25 with Dust and Sea Salt and RHS shows PM2.5 without Dust and Sea salt.

When focusing on Nigeria, the highest concentrations of anthropogenic PM2.5 concentrations of 40–60 μg/m^3^ was centered near 6°N, 7°E with values rapidly falling off with latitude (Figure 2a). When dust and sea salt is included, the entire country has value greater than 40 μg/m^3^ with the highest values of 60–100 μg/m^3^ near 7°N and 13°N (Figure 2b). Figure 2c shows the rapid reduction in latitudinal anthropogenic PM2.5 concentrations (Figure 2c) but with the inclusion of dust and sea salt a peak in PM2.5 concentrations is found near 7°N with increasing PM2.5 concentrations poleward of 11°N (Figure 2d). These high values of PM2.5 for all combined PM sources are considerably higher than the annual average standards of 10.0 μg/m^3^ from the WHO guidelines good air quality. These high PM concentration of PM2.5 arising from anthropogenic and natural sources over Nigeria can be considered unhealthy on an annual basis and is likely to exacerbate pulmonary and respiratory diseases.

The studies on COVID‐19 within Nigeria, as it relates to air pollution has been limited to it's role in reduction of air pollution (Fuwape et al., 2021; Olusola et al., 2021) and modeling (Dansu & Ogunjo, 2021). Because pollution sources vary across Nigeria (natural and anthropogenic), the potential linkages to COVID‐19 are likely to vary. The local sources of PM were examined using low‐cost air quality sensors across seven administrative states. Prior to this time, only estimates from satellites were used for PM monitoring in the region. More importantly temperature, relative humidity, and particulate matter are measured during the Harmatten season during the COVID‐19 pandemic when natural and anthropogenic sources are likely to be found. The specific objectives of the study are: (a) examine meteorological variables of temperature and relative humidity and air quality variables of visibility, PM1 and PM2.5 and PM10 at seven spatially varying locations from 1 November 2020 to 31 March 2021; (b) Examine statistical relationships between COVID‐19 cases/fatalities and meteorological/air quality variables; (c) Determine the sites where the strongest relationships amongst COVID cases/fatalities, air quality, temperature and relative humidity.

## 2 Methodology

### 2.1 Study Area

This study was carried out in seven (7) States from different regions within Nigeria. The demographic and geographic characteristics of the locations are shown in Table 1. Three of the locations‐Kebbi, Kano and Abuja are located in the northern part of the country while the four locations‐Delta, Edo, Osun, and Ibadan are situated in the southern part. Edo has a borderline tropical savanna climate bordering upon a tropical monsoon climate. The weather is uncomfortably hot and humid year‐round, and generally very dull, especially between July and September. Osun is 320 m above sea level with a tropical climate. The precipitation and temperature in Osun state varies between 900 and 1600 mm and 28–35°C annually respectively (Matthew et al., 2015). Oyo State has the same climate as Osun State. Kano is an historical city that has existed for millennia. It is the commercial nerve centre of Northern Nigeria. The city lies to the north of the Jos Plateau, in the Sudanian Savanna region that stretches across the south of the Sahel. Abuja is the capital city of Nigeria. Abuja has a tropical wet and dry climate with three weather conditions annually warm raining season, blistering dry season, and a short harmattan season. The high altitudes and undulating terrain of the FCT act as a moderating influence on the weather of the territory. The climatology of the region has been studied extensively (Olaniran, 1986).

**Table 1:** Demographic and Geographic Information About the Eight Study Locations in Nigeria

Location	Latitude (°N)	Longitude (°E)	Altitude (m)	Population^a^
Kano	11.980	8.480	360	13,076,892
Kebbi	12.308	4.495	237.21	4,440,050
Abuja	8.991	7.384	456	3,564,126
Edo	6.404	5.619	150	4,235,595
Delta	5.537	6.061	150	5,663,362
Osun	7.759	4.603	320	4,705,589
Oyo	7.443	3.900	230	7,840,864

^a^ Estimated population in 2016 (https://nigerianstat.gov.ng/elibrary).

**Figure 3:**
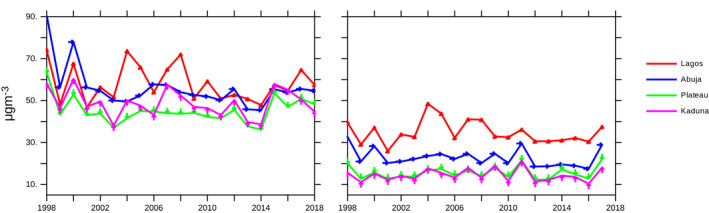
Annual variation of PM2.5 across selected States in Nigeria for (a) PM2.5 with dust and sea salt and (b) without dust and sea salt.

The prevalent air pollution characteristics of the region was determined by the 20‐year mean of PM2.5 distribution using the freely available gridded global PM2.5 data (Hammer et al., 2020). Figure 1 shows the horizontal distribution of PM2.5 with and without dust and sea salt over West Africa. Most parts north of 6°N exhibit low levels of less than 20 μg/m3 (Figure 1a) of PM2.5 concentration annually from natural sources relative to the Guinean coasts which has up to 60 μg/m^3^ (Figure 1a). Generally, the Guinean coast shows up to 10 times more increase in PM2.5 concentrations relative to the northern parts of West Africa (Figure 1c). In contrast, the increase in PM2.5 concentrations from anthropogenic sources (Figures 1b and 1d) are higher than those from natural sources but Nigeria and Niger have the most pronounced concentration of more than 100 μg/m^3^ (Figure 1b). However, since our area of interest for this study is Nigeria, partly because of the good network of monitoring and observing air quality stations, we delve further to take a second preliminary look at the variation of PM2.5 over the country. Figures 2a and 2c suggests the PM2.5 concentration along the Guinean coasts from natural sources is higher than the northern parts of West Africa. This is almost doubled in some areas due to anthropogenic influences (Figure 2d). These high values are considerably higher than the annual average standards of 10.0 μg/m^3^ from the WHO guidelines good air quality. In general, the concentration of PM2.5 arising from anthropogenic sources over Nigeria is very unhealthy to sensitive groups due to aggravation of heart or lung diseases and increased likelihood of respiratory disease. Therefore, the prevalence of high concentration of PM2.5 across Nigeria (Figure 3) already predisposes her citizens to being very vulnerable to infections from COVID‐19. Acute respiratory failure is one of the most common presentation of severe COVID‐19 (Brosnahan et al., 2020). Hence, underlying respiratory ailments coupled with prevalent dust events, will exacerbate COVID‐19 infections in citizens.

### 2.2 Data

**Figure 4:**
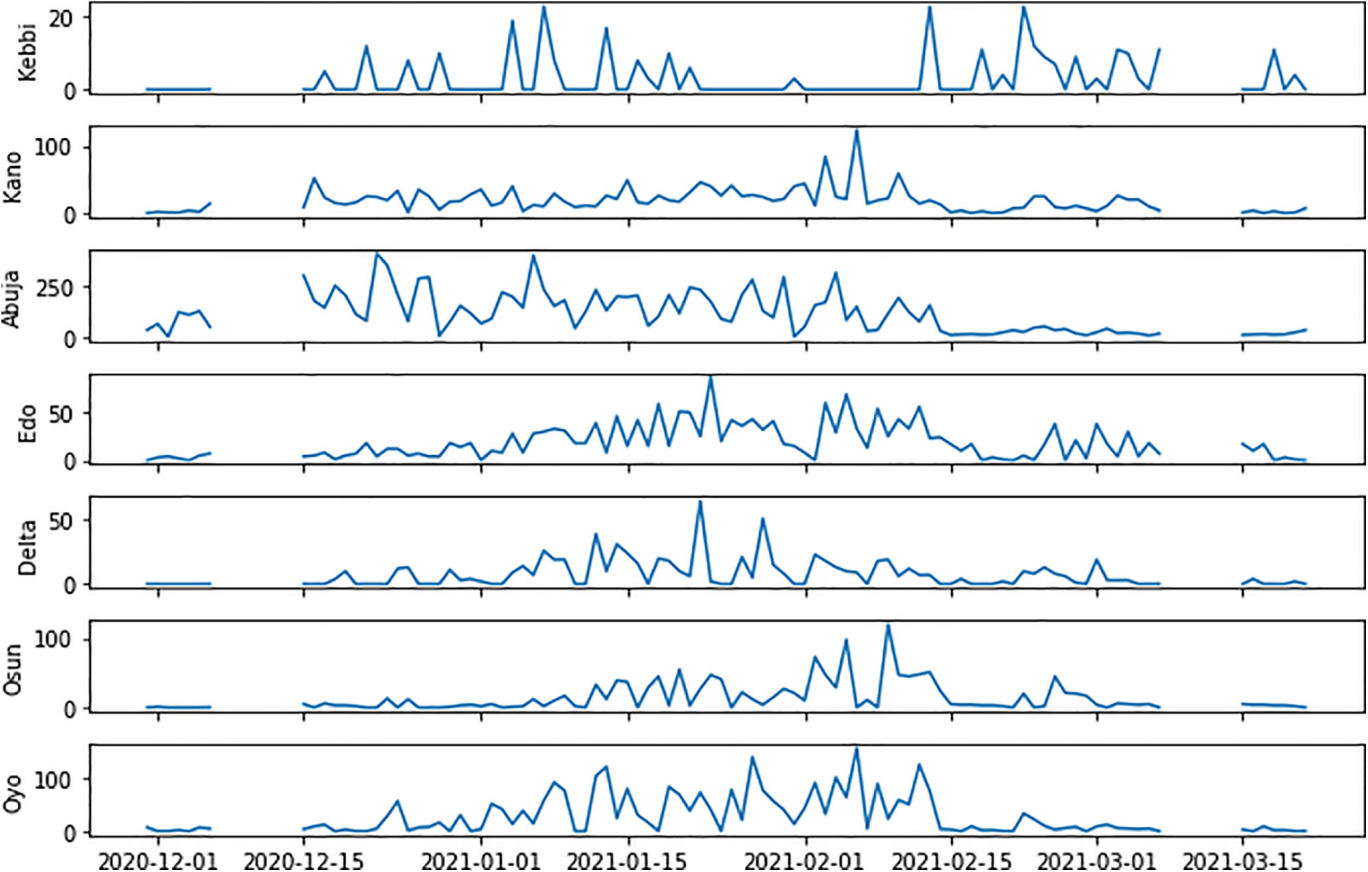
Temporal variation of COVID‐19 cases at different locations for the period under consideration.

To address the lack of PM concentrations in Nigeria, low‐cost air quality stations (called Purple Air stations) were deployed during the fall of 2020. These stations provide a first order approximation of PM2.5 concentrations in locations throughout Nigeria. The stations are geographically located in the central and eastern parts of Nigeria. This data provides a means to evaluate the potential connections amongst of Particulate matter, temperature, relative humidity and COVID‐19 cases and fatalities in seven administrative states of Nigeria. The temporal evolution of COVID‐19 cases in the seven locations are shown in Figure 4. In this work, data from the Purple Air sensors which are low‐cost optical devices for determining particulate matter (Ardon‐Dryer et al., 2020; Liu et al., 2020) from the seven locations in Nigeria were examined. The Purple Air device have two sensors (denoted A and B) that provide estimates of particulate matter (PM1, PM2.5, and PM10), along with relative humidity and temperature. These devices were installed during the onset of the 2020 Harmattan season in Nigeria, and high‐frequency data was averaged to produce daily values. The purple air devices are installed at locations in the 7 Nigerian states shown in Table 1. The geographical distribution of these stations and other information about the device can be found at www.purpleair.com. Other data used in this study include visibility data, particulate matter, COVID‐19 mortality data, and annual particulate matter data. The sources of these data are stated in Table 2. All the data were sampled daily except the annual PM2.5 data.

**Table 2:** Sources of Data Used in This Study

Data	Source
COVID‐19 new cases and mortality	Nigeria Centre for Disease Control (https://ncdc.gov.ng/)
Particulate matter (PM1.0, PM2.5, PM10.0) and atmospheric parameters(temperature and relative humidity)	Purple Air (purpleair.com)
Visibility data	Nigeria Meteorological Agency (NiMet)
Annual PM2.5 data	Atmospheric composition analysis group, Washington University St Louis (https://sites.wustl.edu/acag/datasets/surface-pm2-5/)

### 2.3 Correlation Analysis

The Spearman's correlation coefficient (r) is defined as:

(1) $$\rho =1-\frac{6\sum d_{i}^{2}}{n\left(n^{2}-1\right)}$$

The values of *ρ* within the range −1 ≤ *ρ* ≤ 1 is the Spearman's correlation coefficient, *d*_*i*_ the difference between the two ranks of each observation, and *n* is the number of observations. For comparison, the Pearson correlation was also considered. It is defined as:

(2) $$r=\frac{\sum \left(x_{i}-\bar{x}\right)\left(y_{i}-\bar{y}\right)}{\sqrt{\sum {\left(x_{i}-\bar{x}\right)}^{2}\sum {\left(y_{i}-\bar{y}\right)}^{2}}}$$

where $\bar{y}$ represent the mean value.

## 3 Results

**Figure 5:**
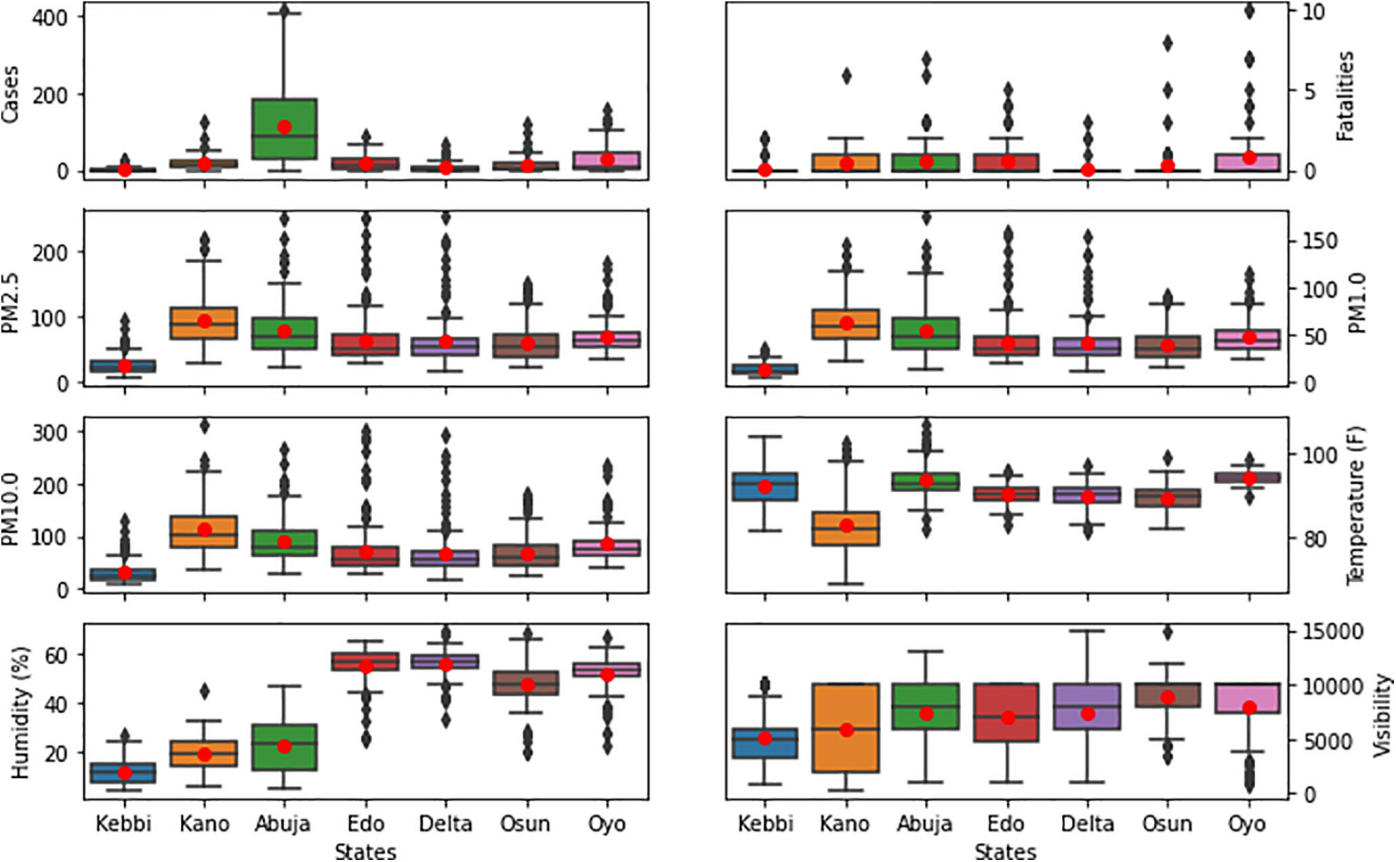
Boxplot showing the variation of the different predictive factors considered in this study.

The temporal variation of COVID‐19 cases within the locations under consideration is shown in Figure 4. Consistent variations were observed between 15 December 2020 and 15 February 2021. The daily cases were as high as 250 in Abuja. The statistical variation of COVID‐19 cases and fatality, as well as, particulate matter (PM1.0, PM2.5, PM10.0, Visibility) and atmospheric parameters (temperature and humidity) are shown via boxplot in Figure 5. Abuja, the country's capital, was observed to have the highest number of cases among the locations considered while Kebbi has the lowest mean values. The high number of cases in Oyo state could be attributed to the proximity with the country's commercial nerve centre, Lagos State. The number of fatalities are characterized by a significant number of outliers. Very low numbers of fatalities were observed in Kebbi, Delta, and Osun states. The high interquartile range found in Kano, Abuja, and Edo states is driven by deadlier strains of COVID‐19 from out‐of‐country visitors and returnees during the period. The particulate matters (PM1.0, PM2.5, PM10.0) were found to be higher in the northern states of Kano and Abuja. The high values are as a result of both anthropogenic sources and dust from the Sahara desert, especially the Bodele region (Goudie & Middleton, 2001). The low value of particulate matter in northern Kebbi State can be attributed to its low altitude which enables overpass of Bodele dust and low contribution of anthropogenic sources in the location (ALU, 2018). In the southern states, where the incursion of Sahara dust is limited, the high values of particulate matter has been attributed to anthropogenic activities from gas flaring (Giwa et al., 2019) and biomass burning (Obioh et al., 2013). The temperature variation during the harmattan period for the locations showed little variability from the interquartile range. However, significant temperature swings were observed in Abuja from the outliers. Kano has the lowest mean temperature and highest variation of all the locations considered. The relative humidity for the locations showed latitudinal delineation. The northern locations exhibited low relative humidity values (below 40%) while the southern locations showed high relative humidity values with incidences of low values during the period. Kebbi State has the lowest visibility of all the locations during the period. This is due to its low altitude and topography within the Sokoto river basin (Aliero et al., 2013). The visibility in Oyo state showed incidences of low visibility during the period under consideration.

**Figure 6:**
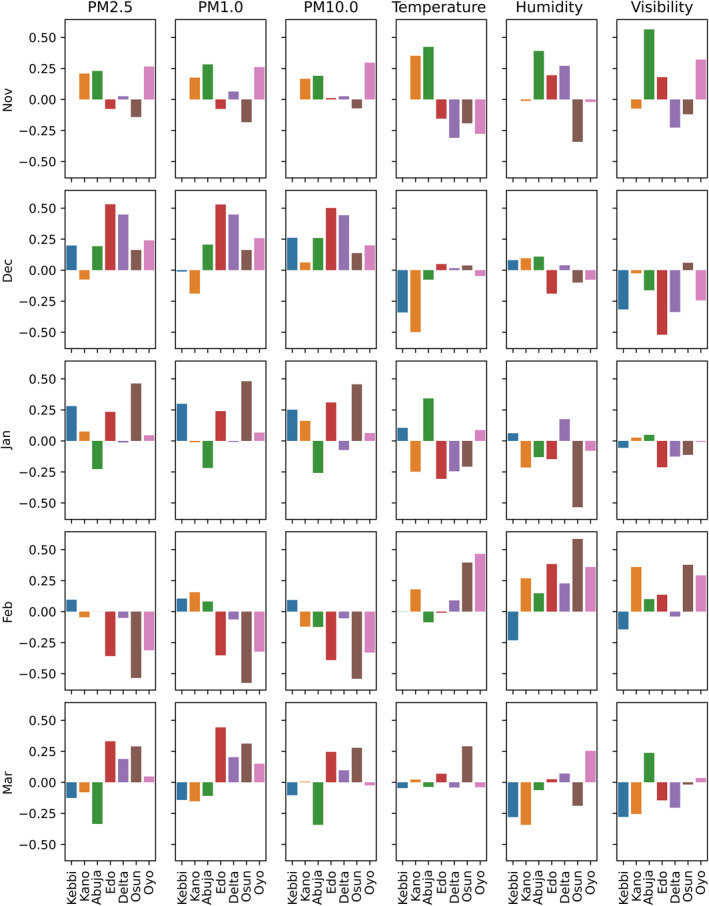
Monthly correlation between COVID‐19 cases and selected parameters.

Monthly correlation between COVID‐19 cases and selected parameters were considered and the results shown in Figure 6. There were no observed COVID‐19 cases in Kebbi State for the month of November, hence, no bar is shown. In November, four states (Kano, Abuja, Delta, and Osun) showed positive correlation between COVID‐19 and particulate matter (PM1.0, PM2.5, PM10.0) while two locations (Osun and Edo) showed negative correlations. The southern locations were found to have negative correlation between COVID‐19 cases and temperature in November while the northern states showed positive correlations. In November, different particulate matter regimes were prevalent in the region, hence, the different correlation results for individual locations. The positive correlation between particulate matter and COVID‐19 cases suggests a plausible relationship in the northern locations (Kano and Abuja). This could be established in December where all locations were found to show positive correlation between particulate matter and COVID‐19 cases with the exception of Kano for PM1.0 and PM2.5. The results from Kano suggest that larger particles play more role in the transmission than smaller particles, however, this is not conclusive. In December, several episodes of Saharan dust incursion reaching the coastal region could be responsible for the positive correlation with COVID‐19 cases (Onyeuwaoma et al., 2015). Furthermore, the northern states showed stronger negative correlations with COVID‐19 cases than the weak positive correlation in the southern states. Moreover, the northern states showed positive correlation between relative humidity and COVID‐19 cases while the southern states exhibit negative correlation with the exception of the Delta state. Generally, COVID‐19 was negatively correlated with visibility in all locations under consideration with the exception of Osun State. The role of particulate matter in the transmission of COVID‐19 was reinforced in January. Particulate matter was found to be positively correlated with COVID‐19 cases in all locations except Abuja and Delta for PM10.0. Temperature and humidity were generally negatively correlated with COVID‐19 cases in January except Kebbi, Abuja, and Oyo (temperature) and Kebbi and Delta (relative humidity). Weak correlations were observed for visibility in January. The southern states showed significantly strong correlations for particulate matter in February. COVID‐19 cases in Kebbi State were found to be positively correlated with all the particulate matter. During February, the role of Saharan dust in the southern states is greatly reduced while biomass burning due to land clearing in preparation for the incoming planting season and gas flaring were the predominant source of particulate matter. The northern state showed weak correlation with PM1.0. In March, there was an inversion, the southern states showed positive correlations between COVID‐19 and particulate matter while the northern states showed negative correlations. With the exception of Kebbi State, relative humidity was found to be positively correlated with the number of cases in February. However, in March most of the locations reported negative correlation for relative humidity.

Spearman correlation between the selected predictors with the number of cases (Table 3) and number of fatalities (Table 4), as well as, the Pearson correlation of the selected predictors with the number of cases (Table 5) and number of fatalities (Table 6) were also considered for the harmattan period, November to March. The two correlation methods showed weak insignificant correlations between particulate matters (PM1.0, PM2.5, PM10.0) and number of cases in Kebbi and Kano states for the period while the other states showed higher and significant correlations. It was observed that locations in oil producing areas (Edo and Delta) have the highest significant correlation values between the number of cases and particulate matters in the Spearman correlation. However, in the Pearson correlation, Osun State has the highest correlation in the number of cases with particulate matter. Kebbi state did not show any significant correlation with the particulate matters and temperature in both correlation methods. This can be attributed to the peculiar nature of the location within the Sokoto/Rima basin, giving rise to the low altitude and topography of the area (Aliero et al., 2013). However, the low humidity in the region is found to be negatively correlated with the number of cases in both Spearman and Pearson correlation methods. Significant negative correlation between the number of cases and temperature in Kano, Abuja, and Osun States was reported in both correlation methods considered. It was concluded that other exogenous factors are responsible for the reported correlation value in Osun State. Furthermore, significant negative correlations were also observed for Kebbi, Kano, and Osun State with relative humidity in the Spearman method but only Osun State showed significant correlation in the Pearson method. However, three states (Kebbi, Edo, and Delta) in the Spearman correlation method and (Edo and Delta) in the Pearson correlation method have significant negative correlations with visibility. Oyo state did not show any significant correlation with temperature, relative humidity, or visibility. It was inferred that smaller particulate matter sizes from biomass burning and gas flaring played a crucial role in the transmission of COVID‐19 virus. Hence, the high correlation in southern states where these acts are prevalent. Generally, particulate matters were observed to be positively correlated with the number of cases while relative humidity is negatively correlated.

**Table 3:** Spearman Correlation Between COVID‐19 Cases and Particulate Matter (PM1.0, PM2.5, PM10.0)/Atmospheric Parameters (Temperature and Humidity) at the Different Locations Under Consideration

Location	PM2.5	PM1.0	PM10.0	Temperature	Humidity	Visibility
Kebbi	0.06	0.01	0.07	−0.08	−0.22^a^	−0.22^a^
Kano	0.02	−0.03	0.01	−0.32^a^	−0.19^a^	0.07
Abuja	0.33^a^	0.33^a^	0.23^a^	−0.31^a^	−0.07	−0.06
Edo	0.47^a^	0.48^a^	0.44^a^	−0.08	−0.02	−0.32^a^
Delta	0.44^a^	0.44^a^	0.43^a^	−0.02	0.01	−0.47^a^
Osun	0.41^a^	0.42^a^	0.41^a^	0.20^a^	−0.30^a^	−0.02
Oyo	0.27^a^	0.28^a^	0.22^a^	0.03	−0.04	−0.03

^a^ Significant values at 95% confidence interval.

**Table 4:** Spearman Correlation Between COVID‐19 Fatalities and Particulate Matter (PM1.0, PM2.5, PM10.0)/Atmospheric Parameters (Temperature and Humidity) at the Different Locations Under Consideration

Location	PM2.5	PM1.0	PM10.0	Temperature	Humidity	Visibility
Kebbi	−0.05	−0.05	−0.05	0.05	−0.02	0.13
Kano	0.03	0.01	0.03	0.27^a^	−0.05^a^	−0.02
Abuja	0.24^a^	0.23^a^	0.27^a^	0.04	−0.17^a^	−0.20^a^
Edo	0.18^a^	0.19^a^	0.18^a^	0.11	−0.05	0.03
Delta	0.06	0.06	0.06	0.11	−0.14	−0.01
Osun	0.24^a^	0.24^a^	0.22^a^	0.11	−0.19^a^	−0.22^a^
Oyo	0.07	0.09	0.05	0.09	−0.06	0.09

^a^ Significant values at 95% confidence interval.

**Table 5:** Pearson Correlation Between COVID‐19 Cases and Particulate Matter (PM1.0, PM2.5, PM10.0)/Atmospheric Parameters (Temperature and Humidity) at the Different Locations Under Consideration

Location	PM2.5	PM1.0	PM10.0	Temperature	Humidity	Visibility
Kebbi	−0.01	0.00	−0.01	−0.03	−0.14	−0.06
Kano	−0.07	−0.11	−0.05	−0.27^a^	−0.13	0.04
Abuja	0.28^a^	0.29^a^	0.20^a^	−0.33^a^	−0.07	−0.06
Edo	0.26^a^	0.29^a^	0.24^a^	−0.10	0.01	−0.38^a^
Delta	0.17	0.18^a^	0.15	−0.03	−0.02	−0.39^a^
Osun	0.28^a^	0.30^a^	0.27^a^	0.18^a^	−0.22^a^	−0.08
Oyo	0.10	0.12	0.08	0.08	0.00	−0.03

^a^ Significant values at 95% confidence interval.

**Table 6:** Pearson Correlation Between COVID‐19 Fatalities and Particulate Matter (PM1.0, PM2.5, PM10.0)/Atmospheric Parameters (Temperature and Humidity) at the Different Locations Under Consideration

Location	PM2.5	PM1.0	PM10.0	Temperature	Humidity	Visibility
Kebbi	−0.07	−0.05	−0.08	0.06	−0.05	0.19
Kano	−0.04	−0.04	−0.07	−0.08	−0.03	0.07
Abuja	0.20^a^	0.19^a^	0.22^a^	0.09	−0.12	−0.18^a^
Edo	0.08	0.10	0.08	0.16	0.00	−0.03
Delta	0.00	0.00	0.00	0.10	−0.06	0.02
Osun	0.17^a^	0.17^a^	0.15	0.11	−0.15	−0.07
Oyo	−0.02	−0.01	−0.02	0.06	0.09	0.07

^a^ Significant values at 95% confidence interval.

The observed correlation between COVID‐19 cases and atmospheric parameters (temperature and humidity) and particulate matter (PM1.0, 2.5, 10.0) during the harmattan season in Nigeria could be attributed to a combination of socio‐economic, demographic, and environmental factors (Table 3). In Kebbi State, the non‐significant correlation between the number of cases and atmospheric factors can be attributed to the low topography. In this location, the dust pattern has been shown to differ from that of the surrounding region with distinct particle size distribution (Essienimo et al., 2015). The low infection rate, number of cases, and correlation values within this region can be attributed to the distance to any international airport, sparse housing, use of hijabi by women, and low influx of foreigners. Furthermore, the state is largely agrarian in nature with little social interaction within the city centre. The population density of Kano has been reported to be between 142.2 km^2^ in the rural regions to 30, 658 km^2^ in the capital city. The location is subjected to regular dust intrusion from the Bodele depression during the harmattan season (Ogunjobi et al., 2012). From our perspective, the cultural inclination of the region is largely responsible for the non‐significant low correlation between COVID‐19 cases and particulate matter. The State has a female population of about 50%, most of whom practice Islam (Ibrahim, 2014). A tenet of the Islamic faith is the wearing of *hijab*, which is strictly enforced within the northern region of Nigeria. The wearing of hijab act as a nose cover for the women. Hence, this limits the susceptible population to those who do not use hijab. Furthermore, there has been reports of under‐ascertainment within the State (Musa et al., 2021).

The correlation between the selected predictors and number of fatalities was also reported for Spearman (Table 4) and Pearson (Table 6). As in the number of cases, positive correlations are reported between the number of fatalities and particulate matter (PM1.0, PM2.5, PM10.0) in the southern states except Oyo State in the Pearson correlation. This supports the findings that increased annual concentration of PM2.5 from anthropogenic sources along the southern states (Figures 2 and 3) could pose high risks of respiratory diseases to sensitive groups. Significant negative correlation was only observed for temperature in Kano, however it was not significant in the Pearson method. This suggests that temperature is a critical factor in the transmission and fatality due to COVID‐19 in Kano. As opposed to the number of cases, positive correlations were observed between temperature and number of fatalities except for Kano State in the Pearson method. In all locations considered, a reduction in relative humidity values was found to correspond to an increase in fatalities. However, only correlation values for Kano, Abuja, and Osun states were found to be significant. Kano and Osun states showed significant negative correlations for both number of cases and number of fatalities from COVID‐19. The two correlation method largely agree except in Kano and Oyo State. The correlation values were found to be lower in Pearson method compared to the Spearman method. Smaller correlation values in the Pearson method can be attributed to outliers (zero values) and the assumption of linear relationship between the variables (Hauke & Kossowski, 2011).

The significant positive correlations in the southern locations (Edo, Delta, Osun, and Oyo) as well as the country's capital (Abuja) could be attributed to the socio‐economic factors. The highly youthful and energetic within the densely populated State's capital is a contributing factor to the spread of the virus in the southern part of Nigeria (Amoo et al., 2020). There is no enforced wearing of hijab, gender segregated transport system, and restricted women activity as obtained in Kano. During the harmattan season, dust from the Bodele region mixed with biomass burning in preparation for the farming season and pollution for industrial and vehicular activities provide an active carrier of the virus to the densely packed and interacting population. Contributing factors to the high correlation in Abuja, Edo, and Delta States include the inflow of infected citizens through the international airports in those locations, as well as their economic indices (Onafeso et al., 2021). Compared to the northern states, the southern states have a vibrant night life which defies government imposed restrictions. This extends the exposure time of the citizen to aerosol borne virus infection. The significant positive correlation between COVID‐19 casualties and particulate matter in Abuja, Edo, and Osun state could be attributed to demographic, cultural, and health infrastructure. The cultural stigma associated with having the virus prevents infected individuals from seek help until it is late (Nachega et al., 2021). In Osun State, with the highest life expectancy in the country, demographic plays a stronger role in the number of casualties recorded (https://nigerianstat.gov.ng/download/952). Furthermore, the state has a doctor to population ratio of about 21,000, hence, the health care system is grossly inadequate for the location (Ajala et al., 2005).

## 4 Conclusion

The spread of the COVID‐19 virus has been attributed to many factors, atmospheric conditions being one of them. It is imperative to investigate the possible relationship between COVID‐19 and atmospheric parameters (temperature and humidity). This will help in mitigating and controlling the spread of the virus within the general populace. In this study, we aim to determine the relationship between COVID‐19 and (a) atmospheric parameters ‐ temperature and humidity, and (b) particulate matters ‐ PM1.0, PM2.5, PM10.0, and visibility, in seven different locations within Nigeria. Results obtained showed that periods of high dust incursion into the country corresponds to the time frames of high number of COVID‐19 cases. Specific local climate such as topography and industrialization were found to contribute to the correlation values obtained on a monthly scale. Generally, during the harmattan season, significant positive correlation values were obtained between COVID‐19 cases and particulate matters in the southern part of the country including Abuja. The significant positive correlation between particulate matters and COVID‐19 cases and fatality will be useful in the control and mitigation of the pandemic spread within localities.

## Conflict of Interest

The authors declare no conflicts of interest relevant to this study.

## Data Availability

All data used in this study are publicly available. (a) [Dataset] The particulate matter (PM1.0, 2.5, 10.0) and atmospheric parameters (temperature and humidity) were obtained from purpleair.com (https://www.purpleair.com/sensorlist?exclude=true&nwlat=9.37944961738998&selat=5.6830568335046365&nwlng=1.5013524736630188&selng=9.784479996099975&sensorsActive2=604800). (b) [Dataset] The COVID‐19 statistics were downloaded from https://covid19.ncdc.gov.ng/state/.

## References

[gh2305-bib-0001] Abu‐Rayash, A. , & Dincer, I. (2020). Analysis of mobility trends during the Covid‐19 coronavirus pandemic: Exploring the impacts on global aviation and travel in selected cities. Energy Research & Social Science, 68, 101693. 32839706 10.1016/j.erss.2020.101693 PMC7365059

[gh2305-bib-0002] Ajala, O. , Sanni, L. , & Adeyinka, S. (2005). Accessibility to health care facilities: A panacea for sustainable rural development in Osun state Southwestern, Nigeria. Journal of Human Ecology, 18(2), 121–128.

[gh2305-bib-0003] Aliero, A. , Shehu, K. , Manga, S. , & Bagudo, A. (2013). Prevalence of dermatophytosis among school children in Kebbi state, Nigeria. Equity Journal of Science and Technology, 1(1), 47–51.

[gh2305-bib-0004] Alu, S. E. (2018). Spatial distribution of harmattan dust (aerosol) loading on horizontal visibility over northern Nigeria (Unpublished doctoral dissertation). Federal University Of Technology.

[gh2305-bib-0005] Amegah, A. K. , & Agyei‐Mensah, S. (2017). Urban air pollution in Sub‐Saharan Africa: Time for action. Environmental Pollution, 220, 738–743. 10.1016/j.envpol.2016.09.042 27646170

[gh2305-bib-0006] Amoo, E. O. , Adekeye, O. , Olawole‐Isaac, A. , Fasina, F. , Adekola, P. O. , Samuel, G. W. , & Azuh, D. E. (2020). Nigeria and Italy divergences in coronavirus experience: Impact of population density. The Scientific World Journal. 10.1155/2020/8923036 PMC7262732 32528234

[gh2305-bib-0007] Ardon‐Dryer, K. , Dryer, Y. , Williams, J. N. , & Moghimi, N. (2020). Measurements of PM 2.5 with purpleair under atmospheric conditions. Atmospheric Measurement Techniques, 13(10), 5441–5458.

[gh2305-bib-0008] Armocida, B. , Formenti, B. , Ussai, S. , Palestra, F. , & Missoni, E. (2020). The Italian health system and the Covid‐19 challenge. The Lancet Public Health, 5(5), e253. 32220653 10.1016/S2468-2667(20)30074-8 PMC7104094

[gh2305-bib-0009] Ayinde, K. , Lukman, A. F. , Rauf, R. I. , Alabi, O. O. , Okon, C. E. , & Ayinde, O. E. (2020). Modeling Nigerian Covid‐19 cases: A comparative analysis of models and estimators. Chaos, Solitons & Fractals, 138, 109911. 10.1016/j.chaos.2020.109911 PMC7282783 32536757

[gh2305-bib-0010] Bauer, S. E. , Im, U. , Mezuman, K. , & Gao, C. Y. (2019). Desert dust, industrialization, and agricultural fires: Health impacts of outdoor air pollution in Africa. Journal of Geophysical Research: Atmospheres, 124(7), 4104–4120. 10.1029/2018jd029336

[gh2305-bib-0011] Ben‐Ami, Y. , Koren, I. , Rudich, Y. , Artaxo, P. , Martin, S. , & Andreae, M. (2010). Transport of North African dust from the bodélé depression to the Amazon basin: A case study. Atmospheric Chemistry and Physics, 10(16), 7533–7544.

[gh2305-bib-0012] Brosnahan, S. B. , Jonkman, A. H. , Kugler, M. C. , Munger, J. S. , & Kaufman, D. A. (2020). Covid‐19 and respiratory system disorders. Arteriosclerosis, Thrombosis, and Vascular Biology, 40(11), 2586–2597. 10.1161/ATVBAHA.120.314515 PMC7571846 32960072

[gh2305-bib-0013] CDC COVID‐19 Response Team , Bialek, S. , Boundy, E. , Bowen, V. , Chow, N. , Cohn, A. , Ellington, S. , et al. (2020). Severe outcomes among patients with coronavirus disease 2019 (Covid‐19)—United states, 12 February–March 16, 2020. Morbidity and Mortality Weekly Report, 69(12), 343–346. 32214079 10.15585/mmwr.mm6912e2 PMC7725513

[gh2305-bib-0014] CDC COVID‐19 Response Team , Chow, N. , Fleming‐Dutra, K. , Gierke, R. , Hall, A. , et al. (2020). Preliminary estimates of the prevalence of selected underlying health conditions among patients with coronavirus disease 2019—United states, February 12–March 28, 2020. Morbidity and Mortality Weekly Report, 69(13), 382. 32240123 10.15585/mmwr.mm6913e2 PMC7119513

[gh2305-bib-0015] Ciencewicki, J. , & Jaspers, I. (2007). Air pollution and respiratory viral infection. Inhalation Toxicology, 19(14), 1135–1146. 17987465 10.1080/08958370701665434

[gh2305-bib-0016] Conticini, E. , Frediani, B. , & Caro, D. (2020). Can atmospheric pollution be considered a co‐factor in extremely high level of SARS‐CoV‐2 lethality in northern Italy? Environmental Pollution, 261, 114465. 32268945 10.1016/j.envpol.2020.114465 PMC7128509

[gh2305-bib-0017] Dansu, E. J. , & Ogunjo, S. T. (2021). Analysis of infectious disease problems (Covid‐19) and their global impact. In (Chapter. Dynamics of inter‐community spread of COVID‐19). Springer.

[gh2305-bib-0018] De Longueville, F. , Ozer, P. , Doumbia, S. , & Henry, S. (2013). Desert dust impacts on human health: An alarming worldwide reality and a need for studies in West Africa. International Journal of Biometeorology, 57(1), 1–19. 22552909 10.1007/s00484-012-0541-y

[gh2305-bib-0019] Dominici, F. , Peng, R. D. , Bell, M. L. , Pham, L. , McDermott, A. , Zeger, S. L. , & Samet, J. M. (2006). Fine particulate air pollution and hospital admission for cardiovascular and respiratory diseases. JAMA, 295(10), 1127–1134. 16522832 10.1001/jama.295.10.1127 PMC3543154

[gh2305-bib-0020] Du, Y. , & Li, T. (2016). Assessment of health‐based economic costs linked to fine particulate (PM2.5) pollution: A case study of haze during January 2013 in Beijing, China. Air Quality, Atmosphere & Health, 9(4), 439–445.

[gh2305-bib-0021] EPA, D. (2009). Integrated science assessment for particulate matter. US Environmental Protection Agency Washington. 36630543

[gh2305-bib-0022] Essienimo, A. , Momoh, M. , & Akpootu, D. (2015). Differential particle size distribution of aerosol across North Western region of Nigeria. International Research Journal of Engineering and Technology, 2(9), 555–561.

[gh2305-bib-0023] Fang, L. , Karakiulakis, G. , & Roth, M. (2020). Are patients with hypertension and diabetes mellitus at increased risk for Covid‐19 infection? The Lancet. Respiratory Medicine, 8(4), e21. 32171062 10.1016/S2213-2600(20)30116-8 PMC7118626

[gh2305-bib-0024] Fuwape, I. , Okpalaonwuka, C. , & Ogunjo, S. (2021). Impact of Covid‐19 pandemic lockdown on distribution of inorganic pollutants in selected cities of Nigeria. Air Quality, Atmosphere & Health, 14(2), 149–155. 10.1007/s11869-020-00921-8 PMC7474574 32922563

[gh2305-bib-0025] Gautam, S. (2020). Covid‐19: Air pollution remains low as people stay at home. Air Quality, Atmosphere & Health, 13, 853–857. 10.1007/s11869-020-00842-6 PMC7241861 32837609

[gh2305-bib-0026] Giwa, S. O. , Nwaokocha, C. N. , Kuye, S. I. , & Adama, K. O. (2019). Gas flaring attendant impacts of criteria and particulate pollutants: A case of Niger delta region of Nigeria. Journal of King Saud University‐Engineering Sciences, 31(3), 209–217.

[gh2305-bib-0027] Goudie, A. S. (2014). Desert dust and human health disorders. Environment International, 63, 101–113. 24275707 10.1016/j.envint.2013.10.011

[gh2305-bib-0028] Goudie, A. S. , & Middleton, N. J. (2001). Saharan dust storms: Nature and consequences. Earth‐Science Reviews, 56(1–4), 179–204.

[gh2305-bib-0029] Gulati, P. , Kumar, A. , & Bhardwaj, R. (2021). Impact of Covid19 on electricity load in Haryana (India). International Journal of Energy Research, 45(2), 3397–3409. 10.1002/er.6008 PMC7675338 33230365

[gh2305-bib-0030] Hammer, M. S. , van Donkelaar, A. , Li, C. , Lyapustin, A. , Sayer, A. M. , Hsu, N. C. , & others (2020). Global estimates and long‐term trends of fine particulate matter concentrations (1998–2018). Environmental Science & Technology, 54(13), 7879–7890. 32491847 10.1021/acs.est.0c01764

[gh2305-bib-0031] Han, L. , Zhou, W. , Li, W. , & Qian, Y. (2017). Global population exposed to fine particulate pollution by population increase and pollution expansion. Air Quality, Atmosphere & Health, 10(10), 1221–1226.

[gh2305-bib-0032] Hauke, J. , & Kossowski, T. (2011). Comparison of values of pearson’s and spearman’s correlation coefficient on the same sets of data. Quaestiones Geographicae, 30(2), 87–93.

[gh2305-bib-0033] Heft‐Neal, S. , Burney, J. , Bendavid, E. , & Burke, M. (2018). Robust relationship between air quality and infant mortality in Africa. Nature, 559(7713), 254–258. 10.1038/s41586-018-0263-3 29950722

[gh2305-bib-0034] Ibrahim, A. M. (2014). How far is too far? The facts and figures on human population in Kano state. International Journal of Humanities and Social Science Invention, 3(4), 61–64.

[gh2305-bib-0035] Isphording, I. E. , & Pestel, N. (2021). Pandemic meets pollution: Poor air quality increases deaths by Covid‐19. Journal of Environmental Economics and Management, 108, 102448. 33850337 10.1016/j.jeem.2021.102448 PMC8028850

[gh2305-bib-0036] John Hopkins University . (2021). Covid‐19 dashboard. Retrieved from https://gisanddata.maps.arcgis.com/apps/dashboards/bda7594740fd40299423467b48e9ecf6

[gh2305-bib-0037] Lacey, F. G. , Marais, E. A. , Henze, D. K. , Lee, C. J. , van Donkelaar, A. , Martin, R. V. , & Wiedinmyer, C. (2017). Improving present day and future estimates of anthropogenic sectoral emissions and the resulting air quality impacts in Africa. Faraday Discussions, 200, 397–412. 28598475 10.1039/c7fd00011a

[gh2305-bib-0038] Lee, S. , Meyler, P. , Mozel, M. , Tauh, T. , & Merchant, R. (2020). Asymptomatic carriage and transmission of SARS‐CoV‐2: What do we know? Canadian Journal of Anaesthesia, 1. 10.1007/s12630-020-01729-x PMC7266417 32488493

[gh2305-bib-0039] Liu, X. , Jayaratne, R. , Thai, P. , Kuhn, T. , Zing, I. , Christensen, B. , et al. (2020). Low‐cost sensors as an alternative for long‐term air quality monitoring. Environmental Research, 185, 109438. 32276167 10.1016/j.envres.2020.109438

[gh2305-bib-0040] Luo, C. , Mahowald, N. M. , & Del Corral, J. (2003). Sensitivity study of meteorological parameters on mineral aerosol mobilization, transport, and distribution. Journal of Geophysical Research, 108(D15), 4447. 10.1029/2003JD003483

[gh2305-bib-0041] Magazzino, C. , Mele, M. , & Schneider, N. (2020). The relationship between air pollution and Covid‐19‐related deaths: An application to three French cities. Applied Energy, 279, 115835. 32952266 10.1016/j.apenergy.2020.115835 PMC7486865

[gh2305-bib-0042] Marone, A. , Kane, C. T. , Mbengue, M. , Jenkins, G. S. , Niang, D. N. , Drame, M. S. , & Gernand, J. M. (2020). Characterization of bacteria on aerosols from dust events in Dakar, Senegal, West Africa. GeoHealth, 4(6), e2019GH000216. 10.1029/2019GH000216 PMC7262684 32490303

[gh2305-bib-0043] Matthew, O. J. , Abiodun, B. J. , & Salami, A. T. (2015). Modelling the impacts of climate variability on crop yields in Nigeria: Performance evaluation of RegCM3‐GLAM system. Meteorological Applications, 22(2), 198–212.

[gh2305-bib-0044] Musa, S. S. , Zhao, S. , Hussaini, N. , Zhuang, Z. , Wu, Y. , Abdulhamid, A. , & He, D. (2021). Estimation of Covid‐19 under‐ascertainment in Kano, Nigeria during the early phase of the epidemics. Alexandria Engineering Journal, 60(5), 4547–4554.

[gh2305-bib-0045] Nachega, J. B. , Atteh, R. , Ihekweazu, C. , Sam‐Agudu, N. A. , Adejumo, P. , Nsanzimana, S. , & others (2021). Contact tracing and the Covid‐19 response in Africa: Best practices, key challenges, and lessons learned from Nigeria, Rwanda, South Africa, and Uganda. The American Journal of Tropical Medicine and Hygiene, 104(4), 1179. 33571138 10.4269/ajtmh.21-0033 PMC8045625

[gh2305-bib-0046] Obioh, I. , Ezeh, G. , Abiye, O. , Alpha, A. , Ojo, E. , & Ganiyu, A. (2013). Atmospheric particulate matter in Nigerian megacities. Toxicological and Environmental Chemistry, 95(3), 379–385.

[gh2305-bib-0047] Ochei, M. , & Adenola, E. (2018). Variability of harmattan dust haze over northern Nigeria. Journal of Pollution, 1(2), 1–8.

[gh2305-bib-0048] Ogunjobi, K. O. , Oluleye, A. , & Ajayi, V. O. (2012). A long‐term record of aerosol index from TOMS observations and horizontal visibility in Sub‐Saharan West Africa. International Journal of Remote Sensing, 33(19), 6076–6093.

[gh2305-bib-0049] Okeahialam, B. N. (2016). Article commentary: The cold dusty harmattan: A season of anguish for cardiologists and patients. Environmental Health Insights, 10. EHI–S38350. 10.4137/EHI.S38350 PMC5004994 27594787

[gh2305-bib-0050] Olaniran, O. J. (1986). On the classification of tropical climates for the study of regional climatology: Nigeria as a case study. Geografiska Annaler‐Series A: Physical Geography, 68(4), 233–244.

[gh2305-bib-0051] Olusola, J. A. , Shote, A. A. , Ouigmane, A. , & Isaifan, R. J. (2021). The impact of Covid‐19 pandemic on nitrogen dioxide levels in Nigeria. PeerJ, 9. 10.7717/peerj.11387 PMC8112247 34012730

[gh2305-bib-0052] Onafeso, O. D. , Onafeso, T. E. , Olumuyiwa‐Oluwabiyi, G. T. , Faniyi, M. O. , Olusola, A. O. , Dina, A. O. , & Adagbasa, E. (2021). Geographical trend analysis of Covid‐19 pandemic onset in Africa. Social Sciences & Humanities Open, 4(1), 100137. 34173513 10.1016/j.ssaho.2021.100137 PMC7931739

[gh2305-bib-0053] Onyeuwaoma, N. D. , Nwofor, O. K. , Chineke, T. C. , Eguaroje, E. O. , & Dike, V. N. (2015). Implications of MODIS impression of aerosol loading over urban and rural settlements in Nigeria: Possible links to energy consumption patterns in the country. Atmospheric Pollution Research, 6(3), 484–494.

[gh2305-bib-0054] Pavilonis, B. , Ierardi, A. M. , Levine, L. , Mirer, F. , & Kelvin, E. A. (2021). Estimating aerosol transmission risk of SARS‐CoV‐2 in New York city public schools during reopening. Environmental Research, 195, 110805. 33508262 10.1016/j.envres.2021.110805 PMC7835536

[gh2305-bib-0055] Sufiyan, I. , Mohammed, K. , Bello, I. E. , & Zaharadeen, I. (2020). Impact of harmattan season on human health in Keffi, Nasarawa state, Nigeria. Matrix Science Medica, 4(2), 44.

[gh2305-bib-0056] Todd, M. C. , Washington, R. , Martins, J. V. , Dubovik, O. , Lizcano, G. , M’bainayel, S. , & Engelstaedter, S. (2007). Mineral dust emission from the Bodélé depression, northern Chad, during BoDex 2005. Journal of Geophysical Research, 112(D6), D06207. 10.1029/2006JD007170

[gh2305-bib-0057] Toure, N. O. , Gueye, N. R. D. , Mbow‐Diokhane, A. , Jenkins, G. S. , Li, M. , Drame, M. S. , & Thiam, K. (2019). Observed and modeled seasonal air quality and respiratory health in Senegal during 2015 and 2016. GeoHealth, 3(12), 423–442. 32159028 10.1029/2019GH000214 PMC7038905

[gh2305-bib-0058] Van Donkelaar, A. , Martin, R. V. , Brauer, M. , & Boys, B. L. (2015). Use of satellite observations for long‐term exposure assessment of global concentrations of fine particulate matter. Environmental Health Perspectives, 123(2), 135–143. 25343779 10.1289/ehp.1408646 PMC4314252

[gh2305-bib-0059] Wang, D. , Hu, B. , Hu, C. , Zhu, F. , Liu, X. , Zhang, J. , & Xiong, Y. (2020). Clinical characteristics of 138 hospitalized patients with 2019 novel coronavirus–infected pneumonia in Wuhan, China. JAMA, 323(11), 1061–1069. 32031570 10.1001/jama.2020.1585 PMC7042881

[gh2305-bib-0060] Washington, R. , Todd, M. , Middleton, N. J. , & Goudie, A. S. (2003). Dust‐storm source areas determined by the total ozone monitoring spectrometer and surface observations. Annals of the Association of American Geographers, 93(2), 297–313.

[gh2305-bib-0061] Washington, R. , & Todd, M. C. (2005). Atmospheric controls on mineral dust emission from the Bodélé depression, Chad: The role of the low level jet. Geophysical Research Letters, 32(17), L17701. 10.1029/2005GL023597

[gh2305-bib-0062] Zhang, X. , Ji, Z. , Yue, Y. , Liu, H. , & Wang, J. (2020). Infection risk assessment of Covid‐19 through aerosol transmission: A case study of south China seafood market. Environmental science & technology. 10.1021/acs.est.0c02895 32543176

[gh2305-bib-0063] Zoran, M. A. , Savastru, R. S. , Savastru, D. M. , & Tautan, M. N. (2020). Assessing the relationship between surface levels of PM2.5 and PM10 particulate matter impact on Covid‐19 in Milan, Italy. The Science of the Total Environment, 738, 139825. 32512362 10.1016/j.scitotenv.2020.139825 PMC7265857

